# Celiac Disease in Adult Patients: Specific Autoantibodies in the Diagnosis, Monitoring, and Screening

**DOI:** 10.1155/2014/623514

**Published:** 2014-04-03

**Authors:** Evagelia Trigoni, Alexandra Tsirogianni, Elena Pipi, Gerassimos Mantzaris, Chryssa Papasteriades

**Affiliations:** ^1^Department of Immunology-Histocompatibility, “Evangelismos” General Hospital, 10676 Athens, Greece; ^2^Department of Dermatology, University of Lübeck, 23562 Lübeck, Germany; ^3^Department of Gastroenterology, “Evangelismos” General Hospital, 10676 Athens, Greece

## Abstract

The increasing prevalence of celiac disease (CD), especially in adults, its atypical clinical presentation, and the strict, lifelong adherence to gluten-free diet (GFD) as the only option for healthy state create an imperative need for noninvasive methods that can effectively diagnose CD and monitor GFD. * Aim*. Evaluation of anti-endomysium (EmA) and anti-tissue transglutaminase IgA (tTG-A) antibodies in CD diagnosis, GFD monitoring, and first degree relatives screening in CD adult patients. * Methods*. 70 newly diagnosed Greek adult patients, 70 controls, and 47 first degree relatives were tested for the presence of EmA and tTG-A. The CD patients were monitored during a 3-year period. * Results*. EmA predictive ability for CD diagnosis was slightly better compared to tTG-A (*P* = 0.043). EmA could assess compliance with GFD already from the beginning of the diet, while both EmA and tTG-A had an equal ability to discriminate between strictly and partially compliant patients after the first semester and so on. Screening of first degree relatives resulted in the identification of 2 undiagnosed CD cases. * Conclusions*. Both EmA and tTG-A are suitable markers in the CD diagnosis, in the screening of CD among first degree relatives, having also an equal performance in the long term monitoring.

## 1. Introduction

Celiac disease (CD) (*coeliac*, from Greek *κοιλιακ* $\overset{´}{\text{o}}$ *ς* koiliakos, “abdominal”) was first described in the second century AD by the Greek physician Aretaeus of Cappadocia as a malabsorptive syndrome with chronic diarrhea [1]. Nowadays it is well known that CD is an immune-mediated systemic disorder elicited by gluten and related prolamins in genetically susceptible individuals and characterized by the presence of a variable combination of gluten dependent clinical manifestations, CD-specific antibodies, HLA-DQ2 or HLA-DQ8 haplotypes, and enteropathy. The typical but not pathognomonic lesions of the small intestinal mucosa resolve with the removal of gluten from the diet [2].

The autoantigen which is also the molecule recognized by anti-endomysium antibodies (EmA) has been identified as the enzyme “tissue transglutaminase” (tTG) [3, 4]. tTG induces the deamidation of gluten peptides and the formation of novel epitopes that, in association with HLA II antigens, induce the antibody response to gliadin and tTG antigens, resulting in the damage of the small intestinal mucosa [5, 6]. Anti-tissue transglutaminase antibodies recognize the same antigen as EmA, from which they differ in terms of detection method. EmA are tested by the indirect immunofluorescence method (IF) and directed against “reticulin-like” fibres in connective tissue around smooth muscle fibres in the oesophagus, liver, stomach, and bladder of monkeys, in the sections of the jejunum and kidneys of rats and in sections of the human umbilical cord (HUC). However, commercially primate GIT tissue is used in almost all (but not exclusively) centers. For the determination of anti-tissue transglutaminase IgA and IgG antibodies, ELISA with human extractive or recombinant transglutaminase is recommended. Both EmA and anti-tissue transglutaminase antibodies are very specific and sensitive [7–9].

Adult patients with CD rarely present with malabsorption related symptoms. Far more commonly they describe nonspecific or subtle gastrointestinal symptoms or they present with extraintestinal manifestations (atypical or silent form); thus they may initially be overlooked [10, 11].

The increasing prevalence of CD, especially in adults, its atypical clinical presentation, and also the lifelong adherence to a gluten-free diet (GFD) as the only option for healthy state create an imperative need for proper immunological tests that can easily, timely, and effectively diagnose CD and monitor GFD [12–16].

## 2. Aim

The aim of the present retrospective study was (a) to evaluate the efficacy of specific autoantibodies in the diagnosis and monitoring of celiac disease in Greek adult patients, where the prevalent diet is the Mediterranean one, mostly based on whole grains and (b) to assess the frequency of undetected celiac disease among the first degree relatives of CD patients.

## 3. Materials and Methods

### 3.1. Patients and Controls

This long term study which took place in the Department of Immunology-Histocompatibility of “Evangelismos” General Hospital of Athens with the cooperation of the Celiac Disease Clinic of the same hospital included the following groups of individuals.

- 70 Greek adult patients, 50 women and 20 men with mean age 39 ± 11.1 years (range: 19–66) who were newly diagnosed with CD. The monitoring of the patients took place on the moment of the diagnosis when they had a regular unrestricted diet and consequently at 6, 12, 24, and 36 months after the initiation of GFD. 51 of the 70 patients (72.9%) followed a strict GFD while the remaining 19 (27.1%) had a partial compliance with GFD.
- 47 first degree relatives (10 parents, 8 siblings, and 29 offspring) of the aforementioned patients, 23 men and 24 women with mean age 24 ± 15.5 years (range: 1–59). In all these family members on gluten-containing diet, the serological tests were performed only once.
- 70 individuals who constituted the control group. 30 of them were patients with inflammatory and noninflammatory diseases of the intestine (8 with Crohn's disease, 7 with ulcerative colitis, 6 irritable bowel syndrome, and 9 with microscopic colitis), 20 women and 10 men with mean age 41 ± 11.3 years (range 21–55) and the remaining 40 were healthy blood donors, 22 women and 18 men with mean age 38 ± 12.1 years (range: 18–58). The control group was serologically tested also once.

No individual in this study had IgA deficiency.

### 3.2. Methods

The antibodies studied were as follows. (a) Anti-endomysium (EmA) which were determined semiquantitative by the technique of indirect immunofluorescence (IIF) using a commercial kit INOVA (NOVA Lite Monkey Oesophagus IFA Kit/Slides, USA) on a 5-*μ*m-thin cryostat section of distal monkey oesophagus as antigen substrate. Patient samples were tested in dilutions ranging from 1 : 5 to 1 : 2560. The antibody titre was defined as the highest sample dilution yielding fluorescence. Titre below 1 : 5 was considered negative. (b) Anti-tissue transglutaminase class IgA (tTG-A) which were assayed using a commercial anti-tTG type IgA ELISA test kit (QUANTA LiteTM, INOVA Diagnostics, USA). The cut-off value provided was 25 U. ELISA was performed in duplicate according to the manufacturer's instruction. Serum samples were kept frozen at −80°C until assays were performed.

The final diagnosis of CD as well as the inflammatory and noninflammatory diseases of the intestine in all these patients was based on currently accepted criteria [17, 18], after thorough clinical and laboratory investigation, including endoscopy and biopsies from the upper and lower gastrointestinal tract. Histological damage observed in the intestinal biopsy samples in CD patients was graded according to Marsh's classification [18]. Moreover, all patients in the study underwent clinical evaluation at each follow-up visit and experienced dietitians assessed their compliance to the GFD, through a standard questionnaire.

### 3.3. Statistics

EmA were expressed using the negative logarithms of measured values while for the comparison of proportions chi-square and Fisher's exact tests were used. Differences in changes of EmA and tTG-A during the follow-up period in total and between the two studied groups were evaluated using repeated measurements analysis of variance (ANOVA) and Bonferroni correction was used. tTG-A was log-transformed for the analysis of variance due to its skewed distribution. Receiver operating characteristic (ROC) analysis was used and area under the curve (AUC), optimal sensitivity, and specificity were determined. All *P* values reported are two tailed. Statistical significance was set at 0.05 and analyses were conducted using SPSS statistical software (version 18.0).

## 4. Results

### 4.1. Evaluation of EmA and tTG-A in CD Diagnosis

The frequency of autoantibodies tested in the control group and in CD patients at the time of diagnosis before they started GFD is presented in Table 1.

All CD patients but 3 presented with positive EmA while increased values of tTG-A were detected in 66 out of the 70 CD patients. Two of the three EmA negative patients were also tTG-A negative while the third one had increased values of tTG-A (155 U). In these three EmA negative patients the small intestinal biopsy showed Marsh I type lesions.

In the control group, however, none had positive EmA whereas 3 individuals showed borderline values of tTG-A (30 U, 32 U, and 28 U, resp.). Two of them were diagnosed with Crohn's disease and the third one with irritable bowel syndrome.

According to the antibodies frequencies found in the studied groups, sensitivity (Se), specificity (Sp), positive (PPV) and negative predictive value (NPV), and the intervals for CD were calculated (Table 2).

As presented in Table 2, EmA showed higher specificity (100.0%), sensitivity (95.7%), PPV (100.0%), and NPV (95.9%) than tTG-A in the diagnosis of celiac disease. Furthermore, their predictive ability for CD diagnosis for CD was slightly better in comparison to the one of tTG-A (*P* = 0.043).

### 4.2. Changes of EmA and tTG-A over Time after Initiation of GFD

**Figure 1:**
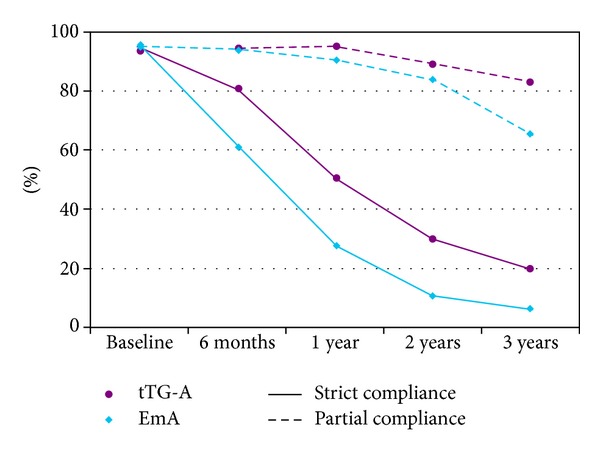
Changes in the percentage of positive samples for EmA and tTG-A over time in strictly compliant and partially compliant patients.

The serological changes over time in all the patients, both strictly and partially compliant, are illustrated in Figure 1 which describes the proportions of positive samples at all follow-up time points. There was a significant change in the proportions of the positive samples over time for both strictly (*P* < 0.001) and partially (*P* = 0.037) compliant patients concerning EmA but for tTG-A the change was significant only for those who had a strict diet (*P* < 0.001).

Compared to partially compliant patients, strict compliance was associated with significantly lower proportions of positive EmA and tTG-A results in the first, second, and third year of the followup (*P* < 0.001). The strictly compliant patients' EmA presented a sharper decline of positive samples proportion (*P* = 0.014) than tTG-A, in the first six months. This decline also continued through the first and second year assessment. On the other hand, for the strictly compliant patients, tTG-A showed a less prominent decrease of positive samples in the first semester (*P* = 0.268) which, however, became significant, continuous, and progressive over time (*P* < 0.001).

More specifically, at the time of diagnosis 96.1% and 94.1% of the fifty-one patients who then started a strict GFD had positive EmA and tTG-A, respectively, while after 6 months of strict GFD 60.8% and 80.4% of them were still positive compared with 27.5% and 51% after 1 year of diet. After 3 years only 3 of the patients (5.9%) had antibody titres over the cut-off level for EmA, while 10 (19.6%) remained with tTG-A positive.

### 4.3. Predictive Capacity of EmA and tTG-A for the Compliance with GFD

**Figure 2:**
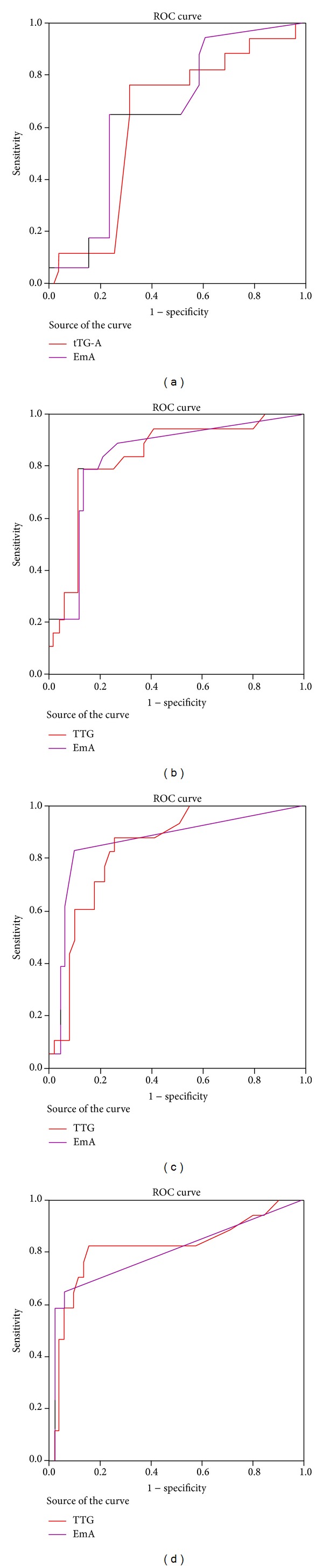
EmA and tTG-A ROC analysis (a) at 6 months after the initiation of GFD, (b) at 1 year after the initiation of GFD, (c) at 2 years after the initiation of GFD, and (d) at 3 years after the initiation of GFD.

The ability of EmA and tTG-A to discriminate the degree of adherence to GFD at the follow-up time points, 6 months, 1, 2, and 3 years was assessed by ROC analysis and is shown in Figures 2(a), 2(b), 2(c), and 2(d), respectively.

We concluded that EmA can assess the degree of compliance with GFD in the first semester from the beginning of the diet, while both EmA and tTG-A have an equal ability to discriminate between strictly and partially compliant patients in the first, second, and third year.

### 4.4. Prevalence of Celiac Disease among the First Degree Relatives

Forty-seven family members of 28 patients with CD underwent serological testing for EmA and tTG-A. Two family members (2/47) (4.2%) had positive EmA while in 5 of them (5/47) (10.6%) increased values of tTG-A were detected.

More specifically, positive EmA was detected in 2 first degree relatives (4.2%), in dilutions 1 : 40 and 1 : 20, respectively, 1 12-year-old girl whose father had CD and 1 18-year-old boy with CD mother. The CD in these family members was confirmed by intestinal biopsy. The two EmA positive individuals also showed high levels of tTG-A (55.0 U and 65.0 U, resp.). However, there were 3 more individuals with tTG-A positive (40.0 U, 60.2 U, and 63.0 U) who were not diagnosed with celiac disease. In these individuals CD diagnosis was excluded by an intestinal biopsy and the fact that they were EmA negative.

## 5. Discussion

Celiac disease in adults presents with a variety of atypical symptoms so there is a need for sensitive and specific serological tests for its accurate and early diagnosis [10]. The only effective treatment for CD is a strict gluten free diet for life [19]. Long term compliance with the GFD is essential to prevent the complications of CD and improve the quality of life [20]. Thus, reliable but also easy applicable markers are needed to monitor patient compliance with these dietary restrictions.

In the present study, which is the first study for celiac disease in Greek adults, the aim was to detect and investigate the specific autoantibodies in the diagnosis, monitoring, and the prognosis of celiac disease.

This study confirms the excellent specificity and sensitivity of EmA (100% and 95.7%, resp.) in CD diagnosis, which is also reported by previous studies [9, 21–27]. We could also recapitulate the high specificity (95.7%) and sensitivity (94.3%) of tTG-A [22, 28–31]. In addition, it was found that EmA predictive value for CD diagnosis was statistically significantly higher compared to tTG-A (*P* = 0.043).

All the three EmA negative patients were relatives of celiac disease patients. At the time of diagnosis two of them had no other clinical symptoms or signs of celiac disease except for mild anaemia which was due to iron deficiency. The third one, however, underwent a thorough endoscopy in order to investigate the cause because of an upper digestive tract bleeding.

Despite the indisputable role of EmA and tTG-A in the diagnosis of CD, the available literature is controversial on their value for assessing compliance with the diet. While some studies have not found the rate of fall of antibody concentration to be a reliable marker of strict adherence to the GFD [32–35], others have found that normalized markers can be useful to confirm GFD, but without concluding which one is the most appropriate [36–40]. In our study fifty-one of the seventeen patients (72.9%) followed a strict GFD while the remaining nineteen (27.1%) had a partial compliance. The serum concentrations of antibodies decreased over time which was inversely correlated with patients' degree of compliance with the diet. Among partially compliant patients, although antibody concentrations also declined, the trends were significantly less pronounced compared with strictly compliant cases.

It was also noticed that, during the first year of a strict compliance with GFD, EmA titres fell more rapidly than tTG-A, whereas in the third year more patients remained having tTG-A positive than EmA despite the fact that they are on a strict GFD. On the other hand, the 19 patients with a partial compliance with GFD presented with a persistence of abnormally elevated antibody concentration that could help in identifying patients with dietary lapses. We propose that EmA can assess the degree of compliance with GFD in the first semester from the beginning of the diet, while both EmA and tTG-A have an equal ability to discriminate between strictly and partially compliant patients in the long term monitoring.

In this study three individuals from the control group as well as three asymptomatic first degree relatives had increased values of tTG-A without having CD. Low levels of tTG-A have been described in a number of conditions unrelated to CD, such as other autoimmune diseases, IBD, infections, tumors, myocardial damage, and liver disorders. These antibodies are not associated with EmA reaction, which explains why EmA has higher reliability for the diagnosis of CD [41–43].

It is well known that CD presents more often among the first degree relatives. In detail, the prevalence of CD among the first degree relatives varies from 2.8% to 8.2% [44–48]. The differences could be partially explained by study methodological differences and the variability of the genetic background of the studied population. In the present study the prevalence of CD among the first degree relatives was 4.2% which is in agreement with the available literature. It is worthwhile mentioning that this prevalence dramatically increases when considering families with two or more cases of CD [49–51].

## 6. Conclusions

The use of serologic markers in celiac is a noninvasive, easily applicable, direct, and reliable practise than can be used for the diagnosis and monitoring of the disease. More specifically, both EmA and the tTG-A are suitable markers in the diagnostic approach of CD. Regarding the ability to discriminate between strictly and partially compliant patients, EmA can assess the degree of compliance with GFD earlier, while both EmA and tTG-A have equal performance in the long term monitoring. We finally recommend the screening for EmA and tTG-A among the first degree relatives.

## Figures and Tables

**Table 1:** Frequency of specific antibodies tested.

	Patients				*P* Pearson's *χ* ^2^ test
	CD patients		Controls		
	*N*	%	*N*	%
tTG-A					
Negative	4	5.7	67	95.7	<**0.001**
Positive	66	94.3	3	4.3	
EmA					
Negative	3	4.3	70	100.0	**<0.001**
Positive	67	95.7	0	0.0	

**Table 2:** Evaluation of autoantibodies tested in CD diagnosis.

	Se (%) (95% CI)	Sp (%) (95% CI)	PPV (%) (95% CI)	NPV (%) (95% CI)
tTG-A	94.3 (86.0–98.4)	95.7 (88.0–99.1)	95.7 (87.8–99.1)	94.4 (86.2–98.4)
EmA	95.7 (88.0–99.1)	100.0 (94.9–100.0)	100.0 (94.6–100.0)	95.9 (88.5–99.1)
